# YouTube and Religion during COVID-19: Analyzing Indonesian Islamic Groups’ Narratives

**DOI:** 10.1007/s10943-025-02462-9

**Published:** 2025-09-30

**Authors:** Achmad Sulfikar, Martin Tanis, Peter Kerkhof

**Affiliations:** 1 Department of Communication Science, Vrije Universiteit Amsterdam, De Boelelaan 1105, 1081 HV Amsterdam, The Netherlands; 2 Department of Communication and Islamic Broadcasting, Universitas Islam Negeri (UIN) Palopo, Palopo, Indonesia

**Keywords:** Covid-19, Social media, YouTube, Advocacy, Online religion

## Abstract

The surge in COVID-19 cases in early 2020 led to the Indonesian government imposing restrictions on public services and religious activities, making YouTube a crucial platform for disseminating pandemic-related information by religious groups. This study examines Indonesian Islamic groups' perspectives on COVID-19 and their use of YouTube. We analyzed the 40 most viewed COVID-19-related YouTube videos from moderate and fundamentalist groups between January and September 2020. The research focused on Islamic groups' beliefs about the pandemic's causes, responses to government policies, and recommendations for dealing with COVID-19. The results showed moderate group videos had more views but fewer social interactions than fundamentalist group videos. Distinct differences were observed, particularly in reactions to government policies, which faced more criticism from fundamentalist videos. Fundamentalist videos portrayed COVID-19 as divine punishment and advocated for an Islamic state as a solution, while moderate groups depicted the pandemic as a test from God and promoted adherence to health guidelines and prayer.

## Introduction

On March 2, Indonesia's President Joko Widodo announced the first two COVID-19 cases, a figure that rapidly grew. On April 2, 2020, Indonesia's death toll rose to 170, with 13 new fatalities, bringing the total number of infections to 1790, while 112 patients were reported to have recovered (The Jakarta Post, 2020). On April 13, 2020, the President declared it a national disaster. In response, the Indonesian government introduced Regulation No. 21 of 2020 on large-scale social restrictions (Peraturan Pemerintah, 2020). This encompassed limiting access to and sometimes closing, government offices, schools, religious institutions, restaurants, and tourist spots.

Religious assemblies were notably restricted. They played a significant role in the disease's spread, evidenced by outbreaks linked to such gatherings in India, Iran, and South Korea (Singh, 2020). Chong et al. (2020) found that a gathering of the Tablighi Jamaat, a major Islamic movement, likely initiated COVID-19 in Malaysia and possibly triggered a second wave in the region. Their belief system perceived COVID-19 not as infectious but as God's will, thus continuing their gatherings and aiding the virus's spread.

However, this was not a universal stance among Islamic groups. The Indonesian Ulema Council (MUI) endorsed government measures. They issued a fatwa on worship during COVID-19 (Majelis Ulama Indonesia, 2020), which outlined worship and burial protocols in line with governmental guidelines, emphasizing the temporary closure of worship places and suspension of mass religious events.

Islam plays a major role in the socio-religious life in Indonesia. Indonesia has the largest number of Muslims in the world; it is estimated that around 229 million Muslims in Indonesia, or 87 percent of the Indonesian population, are Muslim (Statista, 2023). Although the past decade has seen the rise of radical Islamic groups (van Bruinessen, 2013), Indonesian Islam is known for its moderation and inclusiveness, emphasizing harmony and tolerance (Faisal et al., 2022). Despite being a country with a majority Muslim population, Indonesia is home to various Islamic sects and traditions, which contribute to a dynamic religious life (Jubba et al., 2022). Understanding this background is critical for comprehending the interplay between religious dynamics and the societal response to unprecedented events, such as the challenges posed by the COVID-19 pandemic.

Regardless of their stance on government restrictions, numerous religious movements adapted to COVID-19 measures by turning to platforms like YouTube and other social media (Rachmawati et al., 2022; Sukamto & Panca Parulian, 2020). The pandemic brought about an unprecedented need for information and support, but traditional channels were often inaccessible. Throughout the pandemic, information on the virus, its transmission, and preventive measures, including vaccination, evolved rapidly, generating considerable uncertainty (Koffman et al., 2020; Steel et al., 2020). Given their widespread use, social media became a valuable alternative for religious organizations in Indonesia and other countries to address uncertainty and support their congregation.

At the same time, the information provided by religious organizations is not necessarily consistent with scientific insights or supportive of government policies. Given the importance of YouTube in providing information about the pandemic and its potential to influence behavior and, thus, the spread of the virus (Li et al., 2020; Liu, 2022; Parabhoi et al., 2021), in this paper, we examine how Indonesian Islamic groups, both moderate and fundamentalist, have used YouTube to communicate with their followers. We analyze the popularity of these videos and how the messages in these videos convey Islamic groups' responses to government policies, what they consider causes of the pandemic, and what they see as solutions. Since audience reactions play an important role in the spread of videos in YouTube's algorithm, we also examined the relation between video content and (dis)likes and comments.

### YouTube as a COVID-19 Communication Channel: Opportunities and Challenges

Already in 2014, YouTube was an important source of health information when Ebola hit West Africa, with Ebola-related videos being viewed millions of times (Nagpal et al., 2015). In Indonesia, just before the COVID-19 pandemic, YouTube ranked second among the most visited websites, following Google, with 651 million visits per month (Kemp, 2020). Globally, with 2 billion users in 2020, YouTube became an important channel for sharing information about the pandemic (Basch et al., 2020), as illustrated by some viral YouTube videos, such as "The Coronavirus Explained and What You Should Do", with 88 million views. The animated video "COVID-19 Animation: What Happens If You Get Coronavirus" (25 million views) and the video "How wildlife trade is linked to coronavirus" (23 million views) are good examples that illustrate how much YouTube has been used for COVID-19 related information.

With different engagement patterns based on location and content sources, YouTube was crucial in sharing COVID-19 information worldwide. Early on, East Asian countries like Singapore, Hong Kong, Korea, and Thailand led the way in sharing updates as the pandemic first broke out in January 2020 (Tsao et al., 2021). As the situation developed, the USA emerged as a leading source of COVID-19 content, with US-based channels, particularly those focused on straight news and entertainment news, attracting millions of views per video (D’Souza et al., 2020). European countries, including the UK and Canada, also notably produced widely viewed content, often focusing on scientific explanations and public health guidance (Li et al., 2020). While governmental and health organizations like the WHO contributed a smaller share of YouTube's COVID-19 content, their videos were recognized for factual reliability, though they were outpaced by more sensational news sources and consumer-driven content in terms of audience reach and engagement (D’Souza et al., 2020; Li et al., 2020).

YouTube offers a swift channel for sharing information on various topics, but its open nature raises concerns about information accuracy and quality (Bora et al., 2018). In the context of health information, this may result in incorrect or even misleading content. For example, Bora and colleagues found that 23% of Zika virus-related videos were misleading, yet these videos gained more views and shares than accurate ones. A similar situation occurred during the Zika virus crisis, with many video references to conspiracy theories (Nerghes et al., 2018), often similar to conspiracy theories regarding COVID-19; for example, during the COVID-19 crisis, conspiracy theories proliferated around the involvement of figures like Bill Gates, who was accused of using the pandemic to microchip the population through vaccines or to further a depopulation agenda.

While YouTube is an important source of health information, it can also unintentionally or intentionally promote misinformation. Misinformation spreads through mechanisms like clickbait. Clickbait tactics use sensationalized headlines and visuals to attract viewers, often distorting facts to gain attention (Palau-Sampio, 2023). Misinformation on YouTube often mimics legitimate journalism or expert opinions, misleading viewers, especially in health contexts (Calvo et al., 2022). This issue is exacerbated by YouTube's recommendation algorithms, which can create echo chambers that reinforce false beliefs, exposing viewers repeatedly to the same misleading messages (Alimardani & Elswah, 2020). Additionally, it is important to note that access to social media is uneven across different regions due to geographical disparities, digital infrastructure, and local regulations. In rural and low-income areas, limited internet access often restricts engagement, and in certain countries, governmental restrictions further limit access to these platforms (Tan et al., 2020).

### Perspectives of Religious Groups on COVID-19

Religious organizations can potentially serve as influential sources of information for their adherents about health, coping, and personal support. However, their views may differ from official policy and even actively undermine them. The current study examined how Islamic groups have used YouTube to communicate their ideas about COVID-19 to their followers, comparing moderate and fundamentalist Islamic groups' views on the outbreak's causes, their recommendations for addressing it, and their responses to government policies. We also examined audience reactions to the topic (views, comments, replies, likes and dislikes). Below are several religious groups' views on the causes of the COVID-19 pandemic, its treatment, and government policy.

#### Causes of the COVID-19 Outbreak

Next to more earthly explanations for the outbreak of COVID-19, some religious groups proclaimed a more divine cause for the outbreak. For example, a Shia Muslim group in Iraq stated that the outbreak is a 'punishment for gay marriage', and some pastors in the USA believed that COVID-19 is a divine punishment for human sins and a call to renew people's faith (Levin, 2020). A pastor from the Philadelphia Church of God declared that the COVID-19 outbreak was a revelation and a sign that the end of times was near (Dein, 2021). A pastor in Baton Rouge, USA, argued that the virus problem was politically motivated, stating that it was a test of loyalty brought by the Antichrist (Wildman et al., 2020). Thus, in addition to virological or environmental causes, there are numerous examples of religious groups seeking divine explanations for the outbreak of COVID-19 and the resulting pandemic.

#### Recommendations for Dealing with COVID-19

Religious organizations may offer COVID-19 advice. Social distancing, handwashing, and sneezing into elbows are among the COVID-19 recommendations. They also recommended changes in religious activities and the establishment of disaster mitigation systems based on the laws of their religions. For example, due to restrictions on congregational gatherings, several religious groups used videoconferencing and virtual gatherings to reach their followers (Wildman et al., 2020). Muslim groups in Malaysia replaced Friday prayers with Zuhr prayers at home. Christian, Hindu, and Buddhist groups livestream religious festivals and prayers (Tan et al., 2021). Muhammadiyah, one of Indonesia's largest Muslim groups, established the Fiqh Bencana, a disaster management system based on Islamic law (fiqh) and ethics that combines classic Islamic legal principles with modern interpretations to handle modern difficulties. Fiqh Bencana advised Muslims on worship traditions during the COVID-19 pandemic, helping them adapt while remaining true to their faith (Suyadi et al., 2020).

Various religious groups worldwide have demonstrated different approaches to navigating the challenges posed by the pandemic. Jewish and rabbinic groups relaxed the minyan's strict rules for carrying out Kaddish (Cooper, 2021). In Serbia, bishops conducted Holy Mass or Eucharist outdoors, advising the elderly and other vulnerable groups to consider participation carefully. Congregational confessions were temporarily halted. The Cardinal's instructions served as religious dispensations, granting Catholic believers’ forgiveness from their obligation to attend church masses, which were conducted without a congregation. Congregational confessions were suspended, and all diocesan and parish catechetical and pastoral programs, meetings, and events were canceled (Begović, 2020).

#### Responses to Government Policies

During the pandemic, religious organizations were not always supportive of government policies. For example, in the USA, the pastor of Grace Community Church opposed the restrictions, calling the policy a form of fear-mongering that leads to the deprivation of freedom (Perry et al., 2021). On the contrary, leaders of the Islamic community in Bosnia and Herzegovina responded positively to the restrictions by issuing guidelines on the group's activities and actively informing members about the latest pandemic updates (Begović, 2020). Christian church leaders in Ghana supported religious restrictions by encouraging their followers to follow the authorities' advice, conducting health campaigns, and educating them about COVID-19 stigmatization (Osei-Tutu et al., 2021).

### Research Questions

In the current study, we explored the impact of religious narratives on societal perceptions and responses to the COVID-19 pandemic in Indonesia. Understanding these narratives is crucial, as they significantly shape public opinions and behaviors during the pandemic. They also often provide moral and ethical guidance that can significantly influence how communities perceive and respond to crises like the COVID-19 pandemic. Given Indonesia's religious diversity and the global context of COVID-19, we expect a variety of perspectives on the causes of the pandemic, the recommendations for addressing it, and the responses to government policies. This leads us to our first research question: which causes, recommendations, and policy responses regarding COVID-19 were mentioned by Indonesian Islamic groups on their YouTube channels? (RQ.1).

Based on existing literature highlighting ideological differences between moderate and fundamentalist Islamic groups, we anticipate that moderate groups may emphasize conformity to government guidelines and collaborative approaches, while fundamentalist groups may take a more independent stance, perhaps rooted in fundamental religious interpretations. This brings us to the second research question: how did COVID-19-related causes, recommendations, and policy responses differ between moderate and fundamentalist Indonesian Islamic groups? (RQ.2).

Considering the potential impact of religious narratives on viewer engagement, we aim to explore differences in the popularity and social engagement of COVID-19-related videos between moderate and fundamental Islamic groups on YouTube. We anticipate that the nature of the content of YouTube videos may influence engagement levels, which leads to our third research question: what is the difference in popularity (views) and social interaction (comments, replies, likes, and dislikes) between COVID-19-related videos of moderate and fundamental Islamic groups in Indonesia? (RQ.3).

## Method

To address our research questions, we conducted a content analysis of COVID-19-related videos from the YouTube accounts of two Indonesian Islamic groups. The first, Nahdlatul Ulama (NU), is a moderate Islamic religious organization founded in 1926, boasting 40 million members. NU, grounded in traditional Islamic values, supports the current state format suitable for Indonesia's diverse society. It has a rich history of advocating religious pluralism, tolerance, and non-violence (Ismail, 2011). The Nahdlatul Ulama (NU) has historically maintained a close relationship with the Indonesian government, shaping the state ideology of Pancasila and promoting moderate Islam. This collaboration continues today in areas such as education and social welfare, reflecting the complex dynamics of Indonesian politics (Arifianto, 2021). NU maintains its YouTube channels, featuring updates on NU activities, Islamic teachings, social issue talk shows, and live prayer streams with NU leaders and their followers.

We compare NU's videos to those of Hizbut-Tahrir Indonesia (HT), a fundamentalist Islamic organization known for its strict interpretation of Islam and the goal of establishing a global caliphate under its interpretation of Islamic law (Muhtadi, 2009). HT is an Indonesian fundamentalist Islamic group associated with Hizbut Tahrir International, headquartered in London, England. HT initiated its activities in Indonesia in 1983, aiming to establish a religious state governed by Islamic law in various spheres (Osman, 2010). However, in 2017, following controversies in Indonesian society regarding HT's activities, the Republic of Indonesia's government disbanded the organization and designated it as a banned entity (Burhani, 2017).

After HT's official dissolution, its key figures and activists persisted in promoting their Islamist ideology through diverse communication channels: radio, television, social media, seminars, and demonstrations (Fealy, 2020). They employ YouTube channels to spread their views, often featuring clerics and prominent figures as presenters. While HT no longer has an official presence or uses the HT name, former activists, including key figures, still employ HT flags and symbols. Their YouTube content covers religious topics and broader societal concerns, encompassing the COVID-19 pandemic.

In selecting samples of community channels from HT and NU, we focused specifically on channels with COVID-19 content as an initial assessment. We selected three channels from each category based on several criteria: official affiliation with the respective organizations to ensure credibility, relevance of the content to religious and societal responses to the pandemic, and measurable levels of engagement such as number of followers, views, comments, and levels of interaction. These criteria are critical to assessing the reach, influence, and ways in which HT and NU are using their platforms to address the challenges of the pandemic while promoting their respective ideologies and policies.

We choose @KhilafahChannel, @FokusKhilafahChannel, and @MuslimahMediaCenterID from HT. The *Khilafah Channel* defines itself as a forum for truth-telling and Islamic justice. It includes videos of Hizbut Tahrir leaders' speeches, research on Hadith and Quran interpretation, Islamic law, and the Islamic Caliphate. *Fokus Khilafah* presents Islamic facts and ideological disputes. It focuses on HT spokesperson Ismail Yusanto. *Muslimah Media Center* analyzes issues in Indonesia and the Islamic world, offering Islam as a practical and efficient answer. It focuses more on women, families, and Islamic generations than other HT channels.

The three selected channels of the NU group are @NUOnlineID, @NUCHANNEL and @tvnu_id. *NU Online* is the official channel of NU, which claims to provide information on social, national, and religious issues while promoting a moderate approach. Its content includes tutorials on worship, *dhikr*, Islamic studies, shalawat and programs featuring NU scholars. *NU Channel* follows a similar format to *NU Online* but offers a more diverse range of NU-related content. *TVNU Televisi Nahdlatul Ulama* is an extension of the *Lembaga Ta'lif wan Nasyr* (LTN), an institution that falls under NU and is tasked with writing, translating, and publishing books and other information outlets. TVNU is one of LTN's initiatives to disseminate information about various NU activities.

From these six channels, we collected videos that were posted in the period from January 1 to September 30, 2020, by using the keywords "Coronavirus", "COVID-19", "Virus Corona" in Indonesian. In total, we collected 351 videos, 177 videos from HT group, and 174 videos from NU group (see Table 1 below).

**Table 1:** Characteristics of Indonesian Islamic group YouTube channels and number of COVID-19-related videos

YouTube channel	No. of subscribers (k)	Total videos	Total views (m)	COVID-related Videos
*HT group*				
Khilafah channel	79	414	1.9	29
Fokus Khilafah	59	103	973.0 (k)	6
Muslimah media center	109	3153	12.7	142
Total	247	3670	15.6	177
*NU group*				
NU online	21	751	29.0	19
NU channel	591	108	56.1	88
TVNU Televisi Nahdlatul Ulama	120	1012	12.9	67
Total	732	1871	98.0	174

From this list, we selected the top 20 COVID-related videos with the most views from the two groups. Together, the 40 selected videos were viewed 3,504,079 times. The mean number of views per video was 8760 (SD = 116), and the mean length was 30 min (SD = 59 min). The total likes received were 179,917, with a mean of 4497 (SD = 3412), and the total dislikes received were 2491, with a mean of 62 (SD = 76). The mean number of comments per video was 1274 (SD = 1310), with 981% of all comments being replies, with a mean of 125 (SD = 112) per video (see Table 2 below).

**Table 2:** Characteristics of the selected 40 videos

	Mean	SD	Median	Min	Max	Sum
Duration in minutes	30	59	6	1	245	1231
Views	8760	115,777	36,801	20,289	538,950	3,504,079
Likes	4497	3412	4350	358	15,000	179,917
Dislikes	62	76	27	8	298	2491
All Comments	1274	1310	381	11	3366	50,996
Replies	125	112	104	0	519	5001

Videos were classified into one of three categories based on whether they contained content about causes, recommendations, or policy responses. A video was categorized under *causes* if it contained information about the origin or the source of COVID-19, either religious or non-religious. Videos that contained recommendations for dealing with COVID-19, ranging from prayer to health tips, were categorized under *recommendations*. A video fell in the category of *responses to government policy* if it contained content that approved or disapproved government policies or provided alternatives for these policies. Videos could contain content of more than one category; if that was the case, they were classified under more than one category.

Next, to analyze whether the video contained causes, recommendations, or responses to government policies, we labeled the videos as having a regular format (i.e., a video providing information or commentary on a specific topic) or a live streaming format (i.e., the streaming of a live event, live interviews with Q&A with followers using the chat column, live interviews without Q&A session, live prayer with followers). The HT sample consisted of 17 regular videos and three live streaming videos. The NU sample consisted of 14 regular videos and six live streams.

Two coders were involved in coding the videos. To understand the video content in depth, both coders watched the 40 most popular videos (20 from each group) from our initial collection of 351 videos using a three-category scheme (causes, recommendations, and policy responses). After that, the set of videos used for analysis was coded independently by both coders, and any differences between coders were resolved in consultation.

## Results

### Topics of Videos related to COVID-19 on Indonesian Islamic Groups YouTube Channels

Among the 40 videos, 23 sub-topics related to the three main topics. In total, 30 (75%) videos contained recommendations, 24 (60%) had responses to government policy, and nine (23%) referred to the causes of the pandemic. NU had considerably more videos with recommendations (20, 50%) compared to HT (10, 25%), whereas HT videos contained more responses to government policy (14, 35%) than NU videos (10, 25%). Causes of the pandemic were more often referred to in NU videos (6, 15%) than videos linked to HT (3, 8%). In quoting the transcripts of the videos we used the codes HT1—HT20 for videos from the HT group, and the codes NU1-NU20 for videos from the NU group.

### Differences between Videos of the Two Indonesian Islamic Groups about COVID-19

#### Causes

Table 3 below describes the content of the selected videos in more detail. It is important to note that a video may contain more than one category. In terms of the *causes* mentioned in the videos, the HT videos emphasized in three videos (349,463 views) that the consumption of forbidden (“haram”) products had resulted in God's wrath, which led to punishment in the form of viruses coming from the animals that were consumed. The HT videos stated that the Chinese people's habit of eating animals such as dogs, bats, rats, or snakes was the origin of the outbreak and spread of the virus. Even though these foods are *haram* in Islam, the video mentioned that the harmful effects of these foods will also affect those for whom it is not forbidden, as illustrated by the following quote from one of the HT leaders in the video, which was from the time when the outbreak had not yet occurred in Indonesia and therefore focused on what was happening in China:

What happened today was because they ate disgusting and forbidden food by Allah. They ate snakes, rats, dogs, and bats, unfit for human consumption. They often eat the animals alive, showing how gross it is. So, their perverted behavior means justifying Allah's wrath. The Coronavirus outbreak that Allah has sent down today, for which the vaccine has not been found, is part of Allah's wrath. (HT.7)

**Table 3:** Description of topics in COVID-19 videos from the YouTube channel of HT and NU

Categories	HT videos		NU videos	
	Total (*n* = 20)	Number of views (*n* = 906,193)	Total (*n* = 20)	Number of views (*n* = 2,597,886)
*Causes*				
Punishment of China from God	1	278,798	0	0
Unclean and bad food	3	349,463	0	0
Virus from animal to human in China	1	30,318	3	268,409
Destructive capitalist system	1	30,318	0	0
A God test	0	0	4	813,522
*Recommendations*				
Isolation and quarantine	4	250,727	5	737,427
Islamic caliphate government system	8	333,967	0	0
Pray to God	2	66,513	13	2,119,058
Social distancing, washing hands, wearing masks	1	40,470	6	864,974
Healthy lifestyle and healthy environment	2	193,938	4	795,805
Halal and nutritious food	1	40,347	2	120,009
Pray at home	1	40,470	9	1,024,715
Distribute health protection tools	0	0	2	504,136
Keep calm and do not panic	0	0	8	1,057,774
*Responses to government policy*				
Criticism of slow response to COVID-19	5	122,030	1	206,180
Criticism of economic policies during pandemic	3	75,429	0	0
Disagree with the PSBB policy and closure of mosques	2	47,683	0	0
Criticism of ignoring the safety of the people	3	69,462	0	0
Criticism for not preparing a good system in dealing with the pandemic crisis	0	0	2	504,136
Support government policies in dealing with COVID-19	0	0	6	622,737
Support closure of mosques	0	0	3	448,986
Disagree with statements belittling covid	2	43,547	0	0
Disagree with labeling Muslim activities as source of virus spread	1	21,218	0	0

Another example can be found in a video (278,798 views) in which the HT group added that in addition to haram food, God’s anger in the form of a pandemic was caused by the arrogance of the Chinese government and its violence against Muslim minorities in China, such as the Uighurs.

What Allah sent down today in the form of a plague is part of Allah's wrath. We see the Chinese people and communist Chinese rulers who are anti-God. They have not acknowledged the existence of religion, and even their attitude is so barbaric towards religious adherents, especially Muslim, and then they show that attitude with arrogance. (HT.1)

Other examples of a cause mentioned in a video can be found in NU videos that state that God created the coronavirus to test people's faith in Him. In NU's view, God's tests can take any form, including COVID-19. Four videos (813,522 views) from the NU stated that the COVID-19 pandemic is a test for humankind from God. Here is a quote from the video:

Allah created the Coronavirus to test the obedience and patience of His servants to Him. (NU.6)

NU proclaims that COVID-19 is a test, and if humans manage to pass it, their quality of life will be better because they will have experienced overcoming the pandemic. One of the NU leading figures explained the reason for the COVID-19 outbreak as follows:

Allah gave this [COVID-19] to us so we can think new things, produce new culture and produce new traditions. So, we ask Allah that we can accept this test so that we are strong enough to accept the new problems that will arise in our lives today. (NU.4)

#### R*ecommendations*

The NU videos contained more recommendations than the HT group videos. Many recommendations presented in the NU videos revolved around engaging in religious practices (present in 13 videos with 2,119,058 views). In these videos, NU primarily advised their followers to pray to God to overcome the COVID-19 pandemic. Because the NU group saw COVID-19 as a test from God to his servants, it is unsurprising that NU's recommendation to its followers is to pray more to God, as illustrated by one of the quotes:

We pray to Allah to eliminate this coronavirus outbreak from Indonesia and worldwide. What happens to us is Allah's will; therefore, we ask Allah to eradicate this disaster. (NU.2)

The recommendation to followers to pray more to God is conveyed in NU's regular videos and live streaming of Istighosah Kubro. Istighosah Kubro is one of the special religious activities organized by NU and attended by thousands. The ceremony contains readings such as Zikir (praising God), Sholawat (praying) for the Prophet Muhammad, and reciting the Koran. Due to the government's policy of maintaining safe physical distancing, leading to large-scale social restrictions (PSBB), Istighosah Kubro was carried out online by NU.

Next to promoting religious practices, our analysis of NU videos also revealed that NU recommended "non-religiously specific" behaviors such as isolation, social distancing, maintaining hygiene and eating nutritious food:

Not only pray, but we also have to make efforts, eat healthy food, always wash our hands with soap, reduce mobility and social distancing. (NU.12)

The HT group emphasized a political approach as the primary recommendation. Eight videos of HT (333,967 views) stated that running the Khilafah Islamiyah government system is the main way to solve all problems, including the COVID-19 crisis. The HT group proclaimed that the capitalist government system is the basis of human problems. From their perspective, the only solution to those problems is to replace the current government system with the Khilafah Islamiyah. We quote one of the statements:

There is a fault in the capitalist system practiced by the countries' leaders today in the face of COVID-19… let us go back to Islam to successfully deal with the epidemic by running the Islamic caliphate system. (HT.11)

In other recommendation topics, both groups highlighted the importance of isolation and quarantine to prevent the spread of the virus. In videos of NU (5 videos, 737,427 views) and HT (4 videos, 250,727 views), it was stated that these recommendations were based on a Hadith of Prophet Muhammad (Prophetic Sayings) in which he said, "If you hear of an outbreak of plague in a place, do not enter it; but if the plague breaks out in a place while you are in it, do not leave it."

Although both groups recommended isolation and quarantine, they differed in how this health approach should be carried out. NU considered the isolation and quarantine measures essential to be carried out by the Indonesian government. On the other hand, HT stated that these measures would only be effective if carried out in the format of the Bimaristan System. The Bimaristan system is based on historical Islamic practices, particularly the early hospitals in the Islamic world. In this system, health measures such as isolation and quarantine are implemented within the framework of an Islamic state to ensure compliance with Sharia law (Miller, 2006).

#### Responses to Government Policies

This last category consists of videos containing content in which government policies were approved or disapproved or if alternatives for government measures were provided. In the context of this research, we consider criticism as statements that emphasize the weaknesses of government policies in dealing with COVID-19. Disagreements are statements that explicitly reject a government policy. The HT videos contained more elements that could be labeled as responses to government policies than the NU videos. In the HT videos, six subtopic responses included three forms of criticism and three of disagreement. The most prominent criticism from HT was the government's slow response to COVID-19, which was found in five videos (122,030 views), as illustrated by this quote from one of the videos:

The growing number of positive patients infected with the coronavirus is evidence of the failure of the government to raise public awareness. Although President Jokowi imposed the PSBB (Large-Scale Social Restrictions) on March 31, 2020, the government's action is too late. (HT.14)

In other videos, HT assessed that many of the government's economic policies should be criticized during the pandemic. Overall, none of the HT group videos expressed support for government policies.

Looking at the content of the NU videos, we also found criticisms of the government policy, specifically regarding the unpreparedness of the government's health system. However, in their videos, the NU group openly supported the Indonesian government's policies related to COVID-19 in six of the videos (622,737 views). One example of this can be found in a video of NU where one of the officials stated:

Prophet Muhammad, whom Allah sent to bring Islam, stated that this religion is easy. However, if someone burdens himself with this religion, he will be overwhelmed. Fasting does not have to be practised by someone who is sick. Also, the necessity to pray at the mosque can be replaced by praying at home to reduce the spread of the virus. The government's efforts to overcome the virus are a sign that the government loves the people; it cannot be blamed; it must be obeyed. Do not be hateful to the government. (NU.4)

In this video, the NU leader explained that the obligation to practice religion should not burden the people, and that under certain circumstances, worship activities can be carried out according to one's situation and condition. He also encouraged the congregation to comply with government calls and regulations related to COVID-19, as these new measures express the government's commitment to and responsibility for public health.

### Comparison of Popularity and Social Interaction of COVID-19 Videos between HT and NU YouTube Channels

Table 4 (below) shows the popularity and social interaction difference between HT and NU YouTube channels. The total number of views of the 40 videos from both groups is 3,504,079. Videos from NU had more views, which 2,597,886 (range = 20,289 to 538,590; *Median* = 85,184; SD = 141,007) compared to HT, which had 906,193 views (range = 20,566 to 278,798; *Median* = 25,611*; *SD = 62,173). The higher number of views on NU videos mirrors the number of subscribers on the channel compared to the HT videos. The longer duration of the NU's videos is partly explained by the fact that six videos are live streams of NU leaders' worship activities.

**Table 4:** Popularity and social interaction of COVID-19 videos on HT and NU YouTube channels

	Channel	Mean	Median	Sum	SD	Minimum	Maximum
Duration (mins)	HT	16	6	322	32	2	142
	NU	45	10	909	76	1	245
Views	HT	45,309	25,611	906,193	62,173	20,566	278,798
	NU	129,894	85,184	2,597,886	141,007	20,289	538,950
Likes	HT	6285	6500	125,700	1130	4300	8800
	NU	2710	1200	54,217	3988	358	15,000
Dislikes	HT	42	21	849	69	8	298
	NU	82	52	1642	79	12	292
Comments	HT	2158	2555	43,167	1029	171	3087
	NU	141	108	2828	126	11	495
Replies	HT	182	151	3646	130	7	519
	NU	67	70	1355	45	0	153

Although the videos of the NU group had more views, the videos of the HT group received more responses in terms of likes (HT 125,700 and NU 54,217). This is because 17 of the videos are of the regular type (compared to 3 live streaming videos), which contain positions on issues and arguments that invite viewers to be active in commenting on the video or leaving “likes” indicating agreement. Interestingly, when looking at the total number of dislikes, we see a reverse pattern: the total number of dislikes of videos from NU (1642) trumped the dislikes of videos from HT (849).

The selected HT videos received 46,813 comments, far more than the videos of the NU group, which got "only" 4183 comments. The low number of comments on NU videos is consistent with the low number of replies to comments on their videos: The total number of replies to comments on the NU videos was 1355, less than half the number of replies to comments on the HT videos (3646). This difference in the number of comments and replies may be because four NU videos are over an hour long (an average of 163 min per video) and had a live stream format where the (online) audience is praying, which may have been a less inviting context for posting comments or replies.

**Fig. 1:**
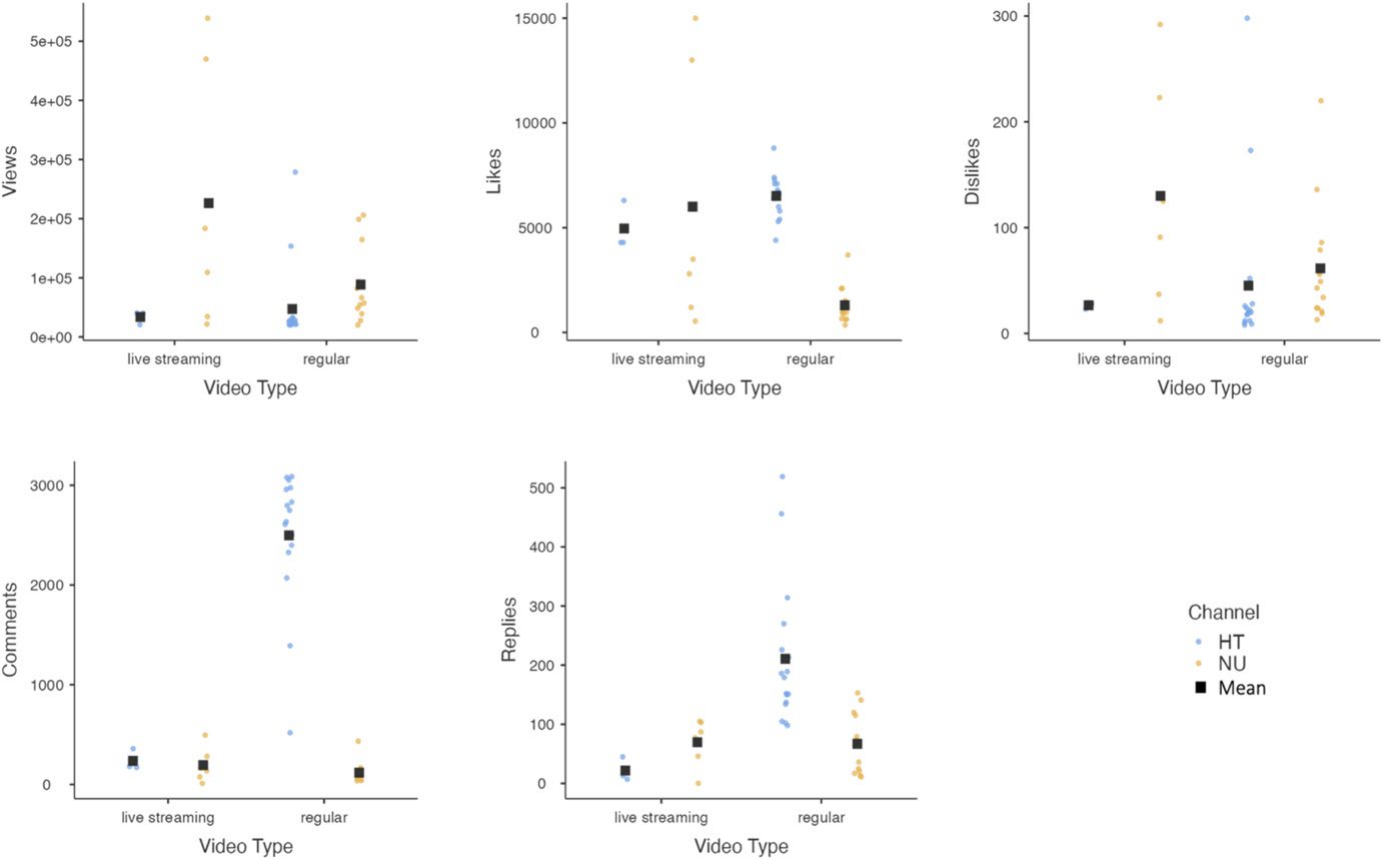
Popularity and social interaction on YouTube channels of Indonesian moderate and fundamentalist Islamic groups based on video types

Figure 1 (below) shows plots illustrating the views and social interactions (including likes, dislikes, comments, and replies) associated with two types of videos—regular and live streaming—from the two different channels.

Looking at the mean values, live streaming videos from the NU channel attracted significant attention, with a mean of 226,312 views. Similarly, regular videos from the same channel also attracted a large audience, with a mean of 88,572 views. This suggests that both types of NU videos are viewed more frequently than their HT counterparts.

Regarding audience engagement through likes and dislikes, we found that regular videos from the HT channel received a higher mean number of likes, specifically 6517, compared to regular NU videos, which received a mean of 1298 likes. However, in the case of live streaming videos, NU outperforms HT with a mean of 6006 likes compared to HT's mean of 4966 likes. Also, HT's regular videos received a mean of 2497 comments and 210 replies, compared to NU's mean of 118 comments and 66 replies.

In summary, while NU's videos, both regular and live streaming, received more views, HT's videos received more likes and generated more conversation, as shown by the higher mean number of comments and replies. This suggests that while NU may have had a wider reach or views, HT's content seems to have engaged its viewers more deeply and encouraged more audience interaction. It is essential to recognize that different types of interactions—such as liking versus commenting—reflect different levels of engagement. Liking a video is a quick, passive interaction, whereas commenting indicates a more active, thoughtful engagement. Additionally, some viewers may engage deeply without leaving any traceable actions, such as praying or reflecting silently, particularly in religious content.

One aspect to consider is the nature of fundamentalist messaging, which often invokes strong emotional responses and fervent reactions from viewers. HT may employ persuasive communication techniques that resonate more intensely with its audience, invoking a sense of urgency and commitment. The heightened engagement observed in HT's content could also be influenced by the ideological alignment between the group and its audience, fostering a stronger sense of communal identity and shared values. Additionally, the distinctive characteristics of fundamentalist groups, including their assertive and uncompromising stances, may contribute to a more passionate and participatory viewer response. This strong emotional engagement is further amplified by emotional contagion on social media platforms. This phenomenon refers to the process by which emotions spread rapidly through social networks, influencing the mood and behavior of others (Lu & Hong, 2022). In the case of HT, the emotionally charged nature of their content likely benefits from this process, significantly enhancing the spread of positive and negative emotions, leading to increased engagement, such as likes, shares, and comments.

## Discussion

The main objective of this paper was to gain insight into how Islamic religious groups in Indonesia discussed COVID-19 on their YouTube channels. More specifically, our study aimed to explain the differences in the YouTube video content of Indonesian moderate (Nahdlatul Ulama, NU) and fundamentalist (Hizbut Tahrir, HT) Islamic groups, focusing on three categories: the proposed causes of the outbreak, the recommendations they offered to deal with it, and the response to the Indonesian government's policies in dealing with the COVID-19 pandemic. We also compared the groups’ videos’ popularity and social interaction to determine audience response and potential spread.

Videos with recommendations were the most popular, followed by videos responding to government policies. The pandemic causes category was the least common in videos from both groups. Videos from the fundamentalist group were more likely to be about responses to government policies, while videos from the moderate group were more likely to be about recommendations (see Table 3). We can draw the following four conclusions based on our examination of the content of the video samples from these two groups.

Our analysis focuses on the differing interpretations of COVID-19's causes by HT and NU. HT attributes the pandemic to unclean food consumption in Wuhan, China, viewing it as a divine punishment for consuming forbidden products. In contrast, NU sees the outbreak as a test from God, an opportunity for progress and spiritual growth. The differing perspectives are based on the groups' distinct religious, cultural, and ideological backgrounds. HT perceives the virus as a divine punishment due to its strict interpretation of religious principles, which views deviations from prescribed practices with severity. However, NU's perspective aligns with its emphasis on spiritual endurance and progress in the face of challenges by framing the pandemic as a test.

Second, regarding recommendations, HT primarily advocates adopting the Khilafah Islamiyah governmental system, believing its laws and values will address issues like COVID-19. NU, meanwhile, encourages increased worship and prayer for strength against the pandemic. Fundamentalist videos often link the pandemic to politics, whereas moderate ones emphasize religious conduct. Nonetheless, both groups endorse essential precautions like social distancing, mask-wearing, and hygiene to curtail the virus's spread.

Third, we found large differences between the two groups in how they respond to government policies. The HT group tends to criticize and disapprove of various government policies, especially the government's slow response to stop the spread of COVID-19. The NU group tends to be more supportive of government policies in their videos, although there is also some criticism in their videos.

Finally, when it comes to viewer engagement and social interaction, our study reveals some interesting contrasts. Notably, NU videos were viewed over 2.5 times more than their counterparts. However, this increased visibility did not translate into higher interaction rates; on the contrary, these videos received nearly 2.5 times fewer likes, generated more than 15 times fewer comments, and received more than 2.5 times fewer replies. The type of video influences the amount of social interaction: Regular videos elicit more reactions from viewers than live-streaming videos.

Several factors contributed to the difference in the number of reactions between the videos. Regular videos, especially from the HT group, contain topics that tend to provoke viewers to comment or dialogue, such as the failure of capitalist countries to deal with COVID-19, criticism of the Indonesian government's pandemic-related policies, or predictions that millions of Indonesians will die because of the failure to deal with COVID-19. In this context, emotions can play an important role in the spread of messages on social media platforms. Emotional content attracts attention and encourages interactions such as likes, shares, and comments, thereby increasing the reach of a message. Emotional contagion significantly enhanced negative emotions spread on social media platforms during the pandemic (Lu & Hong, 2022). In contrast, popular live-streaming videos, especially from NU groups, did not receive many responses or comments because they contained more prayers, religious advice, and explanations of Islamic laws relevant to the pandemic. In addition, the advice and explanations of the laws are given by the leaders of the NU group, who, according to the group's tradition, must be listened to and obeyed by the followers. As a result, most of the audience's responses were repeated prayers and expressions of hope that the pandemic would soon be over.

Our findings reveal varying opinions about the origins of COVID-19 within religious groups, consistent with broader scholarly research on the topic. While some religious narratives have used spiritual terms to describe the global pandemic, such as attributing it to attacks by evil forces and the spirit of the Antichrist (Levin, 2020; Wildman et al., 2020), divine wrath (Østebø et al., 2021), or even apocalyptic events (Dein, 2021), it is important to note that these are only examples and not universally held views. For instance, according to the NU group, the virus may have been transmitted to humans in Wuhan by consuming certain animals, a health-related factor. On the other hand, the HT group attributes the spread of the virus to the food market in China, including the consumption of non-halal food, and even mentions the capitalist political system as the cause of human problems, including the pandemic. This diversity of perspectives emerged in response to the lack of clear information about COVID-19 in the early days of the outbreak (Koffman et al., 2020), which provided an opportunity for different religious perspectives to emerge, some of which included scientific or conventional views alongside spiritual explanations. NU and HT integrated these spiritual and conventional views differently in their responses to the pandemic. NU adopted a balanced approach, combining scientific advice with spiritual guidance and emphasizing that following health protocols was both a civic and religious duty. In contrast, HT leaned more heavily on spiritual and ideological explanations, using the pandemic as a critique of the global capitalist system while downplaying the role of scientific explanations.

Recommendations from the NU group promoting a religious approach such as online Istigosah Kubro as a recommendation to combat COVID-19 are consistent with some of the findings of Ndaluka et al. (2021), who found that religious groups in Tanzania proclaimed that religious activities such as daily prayers and home-based prayers were essential to overcome various social, interpersonal and psychological problems, but could also help overcome health problems.

The close relationship between the NU group and the government is probably one of the best explanations for why the group fully supports the government's policy on COVID-19 (Fealy, 2020). This is best illustrated by the presence of Ma'ruf Amin, a famous Islamic cleric and former leader of the NU group, as the current vice president of Indonesia in an online NU worship video. Stable religious organizations such as NU also determine how they comply with government policy on restrictions; a stable religious organization is integrated into the wider community, has a solid organizational structure and is linked to responsible religious experts (Bawidamann et al., 2021).

The political orientation of the HT group is evident when looking at their recommended solutions and how they respond to government policies related to the COVID-19 pandemic. The HT group's criticisms of government policies cannot be separated from their view that the democratic system is an evil system of government that must be rejected (Burhani, 2017). However, there is an inconsistency between the responses and recommendations of the HT group. In their videos, the HT group disapproves of the government's policy of closing mosques. However, in other videos, they recommend the implementation of isolation and quarantine, including the temporary closure of mosques. As the virus develops, they call for implementing these restrictions based on the Bimaristan system. The implementation of this Khilafah Islamiyah healthcare system is not much different from what the government has ordered. Although the HT group recommends restrictions that are more or less the same as those of the government, these recommendations are based on religious texts and are unrelated to government policy.

The solutions recommended by the HT group are quite similar to the findings of a previous study, which showed that Islamic fundamentalist groups initially opposed the policy of restrictions, but as the spread of the epidemic worsened in Indonesia, these groups began to recommend restrictions themselves (Sukamto & Panca Parulian, 2020). This implies that the impact of the pandemic has changed religious principles in these severe circumstances; the fundamental right to freedom of worship is being restricted, not so much because of government regulations, but because of natural disasters (Alexander, 2020).

The narratives of Indonesia's fundamentalist and moderate Islamic groups on their YouTube channels about the COVID-19 pandemic reflect each group's ideology. As religious groups, it is not surprising that both groups emphasize the role of God in daily life (Alexander, 2020; Pirutinsky et al., 2020). Beyond maintaining contact with followers, YouTube videos discussing the COVID-19 pandemic offer groups a platform to promote their agendas strategically. In our study, we observed how fundamentalist Islamic groups leverage the pandemic to underscore their narratives about the need for an Islamic caliphate system, which they believe is the remedy for Indonesia's myriad social and economic challenges. They present the pandemic as evidence of modern world crises and the shortcomings of capitalist systems. Conversely, moderate Islamic factions use the pandemic to emphasize the significance of religious adherence and moral values in tackling Indonesia's issues. They stress that this pandemic is a divine test, best confronted with patience, unity, and community solidarity.

## Limitations

Some limitations may be addressed in future research. We collected data from January 2020 to September 2020, at the pandemic's beginning. It would be interesting to see if patterns changed as the pandemic continued. Also, while this study focused on video content, we need to know the intentions of the videos. Future research could explore these intentions by interviewing the video makers. Another interesting aspect, but not covered in this study, is how discussions or conversations take place in the comments section of YouTube videos and what the audience's main concerns are in these conversations. It would also be interesting to study the viewers of the videos to see how they perceived them and how they affected their religious practices and ideas during the COVID-19 pandemic.

Additionally, this study does not explore the phenomena of clickbait and echo chambers, which are relevant to understanding how content is consumed and disseminated on social media platforms like YouTube. Clickbait could influence the viewer's choice of content, potentially skewing viewership towards more sensational or emotionally charged videos. Echo chambers may reinforce pre-existing beliefs among viewers, particularly in religious or ideological content, limiting exposure to diverse perspectives. Future research could delve into these aspects to provide a more comprehensive understanding of the digital landscape during the pandemic.

Despite these limitations, the results of our study are insightful and help us to understand how religious groups use YouTube to stay in touch with their followers in times of crisis and the role their videos may play in the dynamics of a pandemic.

## Conclusions

Our study shows that Muslim religious groups tend to prioritize a religious perspective rather than a purely scientific perspective when looking at a crisis. It is crucial to note that our findings pertain specifically to these two polarities of Islam within Indonesia, offering nuanced insights into how they approach and interpret the challenges posed by the COVID-19 pandemic.

Although the cause of COVID-19 can be explained scientifically, religious groups believe that God created all matter in the universe for a specific purpose (Tolmie & Venter, 2021). For moderate groups, praying to God is the main recommendation, along with taking steps to prevent virus transmission according to established health standards. Recommendations from fundamentalist groups focus more on religion-based political elements. This group believes that implementing Islamic politics as a Khilafah Islamiyah government is God's command that must be carried out. For HT, this obedience to God's commandments is the solution to the world's health crisis. Although both groups bring a religious perspective to the debate on COVID-19 policy solutions, this is not to say that the religious perspective crowds out the practical and science-based recommendations proposed by the government. Support for such measures can be found in the videos of both groups. However, the fundamentalist perspective goes hand in hand with strong criticism of government policy, whereas the more moderate perspective does not.

One function of social media is to allow religious organizations to connect with a wider audience and reach people who may not have access to physical religious spaces or gatherings (Kühle & Larsen, 2021). YouTube has become a popular platform for religious organizations, with many offering their followers sermons, lectures, and other religious content. This platform can help religious organizations build and maintain their communities, even in times of crisis, such as the COVID-19 pandemic.

In this study, NU used social media to maintain religious services and teachings for followers who could not attend religious gatherings because of pandemic restrictions. This has helped NU retain its community and provide spiritual and emotional support to its adherents. Religious organizations can promote their messages and engage in public conversation on social media. HT has used YouTube to promote a global Islamic caliphate and oppose the secular Indonesian government. Social media, including YouTube, is vital to religious organizations like HT and NU. These platforms let these groups communicate with their communities, share information, and discuss situations like the COVID-19 outbreak.
